# Effects of Black Wattle (*Acacia mearnsii*) Condensed Tannins on Intake, Protozoa Population, Ruminal Fermentation, and Nutrient Digestibility in Jersey Steers

**DOI:** 10.3390/ani10061011

**Published:** 2020-06-09

**Authors:** Andre S. Avila, Maximiliane A. Zambom, Andressa Faccenda, Maria L. Fischer, Fernando A. Anschau, Tiago Venturini, Rodrigo C. R. Tinini, Jessica G. Dessbesell, Antonio P. Faciola

**Affiliations:** 1 Department of Animal Science, State University of Western Parana, Marechal Candido Rondon 85960-000, Brazil; mazambom@hotmail.com (M.A.Z.); maria.luiza.fischer@hotmail.com (M.L.F.); fernando_anschau@hotmail.com (F.A.A.); venturini_tiago@hotmail.com (T.V.); rodrigotinini@gmail.com (R.C.R.T.); jessicagabidess@gmail.com (J.G.D.); 2 Department of Animal Science, State University of Maringa, Maringa 87020-900, Brazil; andressafaccenda@hotmail.com; 3 Department of Animal Sciences, University of Florida, Gainesville, FL 32611, USA

**Keywords:** additives, digestibility, intake, polyphenols, secondary compounds

## Abstract

**Simple Summary:**

Condensed tannins are plant secondary compounds that can modulate ruminal fermentation by binding to proteins, reducing their ruminal degradation, and also reduce ruminal protozoa, which may improve the efficiency of nitrogen utilization. In this study, we tested increasing levels (0, 5, 10, 15, and 20 g/kg of diet dry matter) of *Acacia mearnsii* condensed tannins in the diets of Jersey steers. Condensed tannins did not affect intake and ruminal protozoa population, but reduced protein digestibility and decreased ruminal pH and acetate proportion. Overall, the tested doses of condensed tannins extract did not improve ruminal fermentation and nutrient digestibility.

**Abstract:**

The objective of this study was to evaluate the effect of inclusion of condensed tannins (CT) from black wattle (*Acacia mearnsii*) on feed intake, ruminal protozoa population, ruminal fermentation, and nutrient digestibility in Jersey steers. Five ruminally-cannulated steers were used in a 5 × 5 Latin square design, with five periods of 20 days each (14 days for diet adaptation and six days for sample collection per period). Treatments were composed of dietary inclusion levels of condensed tannins at 0, 5, 10, 15, and 20 g/kg of diet dry matter. Intakes of dry matter, organic matter, ether extract, crude protein, neutral detergent fiber, and total digestible nutrients were not affected by condensed tannins. The ruminal pH was reduced linearly with tannin levels. Ruminal ammonia nitrogen concentration was not affected by tannins. Tannins reduced the molar proportion of acetate and did not affect the ruminal protozoal population, which might be related to the low doses used. Digestibilities of dry matter, organic matter, and neutral detergent fiber were not altered; however, there was a linear reduction in crude protein digestibility. Based on these results, CT extracts from black wattle are not recommended for improving nutrient utilization in steers at the tested levels.

## 1. Introduction

Tannins are secondary metabolites synthesized by plants [1] and, for a long time, have been considered antinutritional factors due to their adverse effects on feed intake and nutrient utilization by ruminants; however, tannins have been recognized as useful additives that can modulate microbial ruminal fermentation [2].

Condensed tannins (CT) can bind to proteins and can prevent their rapid ruminal degradation, regulating the rate of nitrogen release in the rumen, increasing the flow of essential amino acids to the small intestine [3]. These compounds can also be complexed with other nutrients, such as carbohydrates and minerals [4,5].

Tannins can also modify ruminal fermentation, increasing propionate and decreasing acetate concentrations [6]; however, ruminal changes depend on tannin type, structural chemistry, dose, and basal diet; moreover, some species of microorganisms are more tolerant than others to the effects of tannins [5,7]. Dickhoefer et al. [8] observed a reduction in the acetate to propionate ratio when quebracho tannins (*Schinopsis* sp.) were fed at 40 and 60 g/kg of diet dry matter (DM). However, Aguerre et al. [9] evaluated levels of 4.5, 9, and 18 g/kg of diet DM and did not find effects on pH, total volatile fatty acids, molar proportions of acetate, propionate, and the acetate propionate ratio, whereas a reduction in ruminal ammonia concentration was observed.

On the other hand, high concentrations of CT can cause a reduction in feed intake, nutrient digestion, and, consequently, losses in animal productivity [10]. In addition, high doses may cause serious intoxication problems in ruminants, whose symptoms include increased heart and respiratory rates, anorexia, and ulcers in the rumen and reticulum mucosa [11].

Condensed tannins may also reduce the population of protozoa in the rumen [12] and improve the efficiency of nutrient utilization [13,14] since protozoa prey on ruminal bacteria, which leads to a waste of energy and undesirable recycling of nitrogen in the rumen [15]. Carulla et al. [16] evaluated CT from *Acacia mearnsii* and observed a reduction in Holotrichs with a dose of 25 g/kg of diet DM for sheep, while Perna Junior et al. [17], also using this CT source for dry cows with a dose of 6 g/kg of diet DM, observed a reduction in Isotricha genus by 29% compared to the control treatment.

Condensed tannins of *Acacia mearnsii* have been used as an additive to improve ruminal fermentation and/or nutrient digestibility in ruminants [17,18,19]; however, the results are still conflicting, requiring more studies with its utilization. We hypothesized that CT from *Acacia mearnsii* would reduce ruminal protein degradation and ruminal ammonia concentration and improve ruminal fermentation, as well as nutrient utilization by ruminants. The objective of this study was to evaluate the effect of inclusion of increasing levels of CT extracted from black wattle (*Acacia mearnsii*) on feed intake, ruminal fermentation, ruminal protozoa population, and nutrient digestibility in Jersey steers.

## 2. Materials and Methods

The experiment was carried out at the experimental farm of the State University of Western Parana (Unioeste) located at latitude 24°31′55.3″ S, longitude 54°01′08.0″, and 392 m altitude. The animal experiment protocol was approved by the Unioeste Animal Ethics Committee (protocol 54/16).

Five ruminally-cannulated Jersey steers were used, with a mean bodyweight of 752 ± 34.0 kg. The animals were randomly distributed in a 5 × 5 Latin square, and each experimental period had a duration of 20 days—14 days for diet adaptation and 6 for sample collections. The basal ration was composed of Tifton 85 hay, corn, soybean meal (see Table 1 for chemical composition), and mineral mix. The treatments were inclusion levels of the tannin extract: 0, 6.12, 12.2, 18.4, and 24.6 g/kg DM of the diet.

The product used (Seta RC) was a commercial extract of CT extract from black wattle (*Acacia mearnsii*) (Seta S.A., Estância Velha, RG, Brazil) in a powder form and, according to the manufacturer, with a total concentration of 80.5% of tannins based on DM, 18.0% non-tannins, 1.44% insolubles, 2.11% ash, 59.8 ppm of iron, pH 4.90, and 5.65% humidity. The product was mixed with the other ingredients of the concentrate feed (see Table 2 for chemical composition). Considering the total tannin concentration of the commercial product, the proportion of CT included in the total diet was 0, 5, 10, 15, and 20 g/kg of DM.

Animals were housed in a tie-stall type barn with individual troughs and water ad libitum. The diets were calculated to guarantee the maintenance requirements of the animals according to the NRC(National Research Council) [21] in a forage: concentrate ratio of 60:40 (Table 2), and feed supply was restricted to 22.3 Mcal of metabolizable energy per day, estimated according to the NRC [21]. The diets were offered as total mixed ration twice a day at 06:00 and 16:00 h, in the proportion of 70% and 30% of the total DM, respectively. The animals had access to a resting paddock without pasture from 11:00 a.m. to 14:00. At the beginning and at the end of each experimental period, the animals were weighed before the morning feeding.

From the 15th to the 20th day of the experimental period, the intake was measured by weighing the feed provided and the leftovers. For the determination of nutrient digestibilities, daily samples of feed and leftovers were collected and stored for further analysis. Fecal samples (165 g) were collected direct from the rectum as follows: day 15 (07:50), day 16 (10:00), day 17 (12:00), day 18 (14:00), day 19 (15:50), and day 20 (18:00). The samples were dried in a forced-air oven (55 °C, 72 h), ground to 1-mm sieve screen. Samples were pooled for feed, refusals, and feces, resulting in one sample of each, per animal per period. Samples were analyzed according to Association of Official Analytical Chemists [22] for dry matter (DM, method 934.01), ash (method 938.08), crude protein (CP; method 981.10), ether extract (EE; method 920.85) and determination of neutral detergent fiber (NDF) according to Van Soest et al. [23].

The organic matter (OM) was calculated by the difference between ashes and total DM. Non-fibrous carbohydrates (NFC) were calculated by the equation of Weiss et al. [20]. To estimate daily fecal excretion, the indigestible acid detergent fiber (iADF) was used as the internal indicator. The iADF was determined in feed samples, leftovers, and feces, which were incubated by the in situ method for 240 h, as described by Casali et al. [24]. The total digestible nutrient (TDN) content of the diets was calculated according to the NRC [21].

The analyses of pH, ammonia nitrogen (N-NH_3_), and volatile fatty acids (VFA) in the ruminal content samples were performed on the 20th day of each experimental period at the following times: before feeding (0 h) and 2, 4, 6, and 8 h after feeding. The samples were collected from the dorsal, ventral, and central portions of the rumen, pooled, and strained through four layers of cheesecloth. Immediately after the collection, the pH was measured using a digital pH meter. A 50 mL aliquot of ruminal sample was acidified with 1 mL of sulfuric acid (50%) and frozen (−20 °C) for further N-NH_3_ analysis following the technique adapted by Vieira [25]. For analyses of ruminal VFA (acetate, propionate, and butyrate), 8 mL of ruminal fluid was acidified with 2 mL of metaphosphoric acid 25%. To perform the analysis, high-performance liquid chromatography was used according to the method of Lazaro [26].

For the protozoa analysis, ruminal samples were collected from the dorsal, ventral, and central portions and pooled at 4 h after morning feeding. A 40 mL aliquot of ruminal sample was mixed in equal proportions of formaldehyde for the identification and quantification of ciliates, then 1 mL of the sample was transferred to test tubes, and three drops of Lugol were added, in a modification proposed by D’Agosto and Carneiro [27]. After 15 min, 9 mL of 30% glycerin was added. For quantification, a sample was pipetted into each test tube to fill the Sedgewick-Rafter counting chamber (Labdel^®^-ATC 515050, Microscope World, Carlsbad, CA, USA). A counting grid was used in one of the eyepieces of the microscope (Olympus BX51, Olympus^®^, Hamburg, Germany), and the ciliates present in 50 fields were quantified; after the rotation of the chamber by 180°, further 50 fields were quantified. To calculate the total number of ciliates per mL of content, the values obtained were multiplied by 80 and 20, and these values corresponded to the total chamber surface, counting, and dilution [28]. The protozoa present in each sample were identified based on the criteria described by Ogimoto and Imai [29].

For the statistical analysis, the data were tested for normality using the Shapiro–Wilk test. The intake, digestibility, and protozoa data were analyzed using the mixed procedure of the SAS (Statistical Analysis System, version 9.2). The statistical model used was:

γ_ijk_ = µ +T_i_ + p_j_ + a_k_ + ε_ijk_ (1)

where γ_ijk_ = dependent variable, μ = mean, Τ_i_ = fixed treatment effect (i = 1 to 5), p_j_ = random effect of the period (j = 1 to 5), a_k_ = random effect of the animal (k = 1 to 5), and ε_ijk_ = residual error.

For the evaluation of NH_3_-N, pH, and VFA repeated measures, the analysis was carried out, where the fixed time effect and its interaction with the treatment were included according to the model:

γ_ijkl_ = μ + T_i_ +p_j_+ a_k_+ T_l_ + TD_il_ +ε_ijkl_ (2)

where γ_ijkl_ = observation, μ = mean population, T_i_ = treatment effect (i = 1 to 5), p_j_ = period effect (j = 1 to 5), a_k_ = animal effect (k = 1 to 5), D_l_ = fixed time effect (l = 1 to 5), TD_ij_ = treatment and time interaction and ε_ijkl_ = residual error.

When there was an effect of treatment, orthogonal polynomial contrasts were used to evaluate the response pattern to the increasing levels of CT using the mixed procedure of SAS. Prediction equations were also generated for those significant variables using the regression procedure of SAS. Significance was declared at *p* ≤ 0.05, and trend when 0.05 < *p* ≤ 0.10.

## 3. Results

Intakes of DM, OM, CP, EE, NDF, NFC, and TDN were not affected by CT inclusion (Table 3). Digestibilities of DM, OM, NDF, EE, NFC, and TDN were not affected by CT levels (*p* > 0.10) (Table 4); however, there was a linear reduction in CP digestibility (*p* = 0.03) as CT was included in the animals’ diet.

Regarding the ciliate protozoa (Table 5), there was no effect (*p* > 0.10) on the evaluated genera (*Entodinium, Dasytricha, Isotricha, Charonina, Eremoplast*, and *Metadinium*); thus, a total number of protozoa was not affected by CT dose (*p* > 0.10).

**Figure 1:**
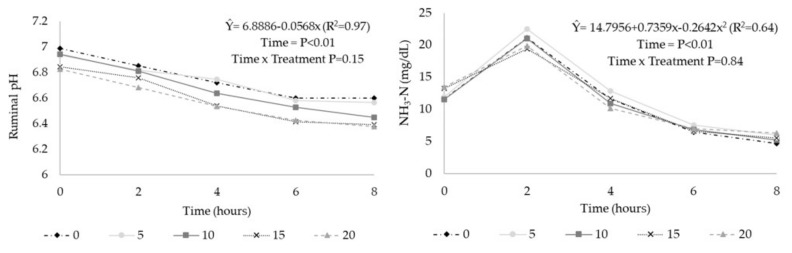
Ruminal values of pH and ammonia nitrogen (NH_3_-N) in steers receiving diets with condensed tannins extract (CT) from *Acacia mearnsii,* as a function of time after feeding. 0 = no CT inclusion; 5 = 5 g of CT/kg of dry matter (DM); 10 = 10 g of CT/kg of DM; 15 = 15 g of CT/kg of DM; 20 = 20 g of CT/kg of DM.

Ruminal pH values were reduced linearly with the inclusion of CT (*p* < 0.01), and there was no effect on ruminal NH_3_-N concentration (*p* > 0.10) (Table 6). In relation to the time after feeding, ruminal pH had a linear reduction (Figure 1; *p* < 0.01), and there was no interaction between CT and time after feeding (*p* > 0.10). For the NH_3_-N contents, there was a cubic effect (Figure 1) in relation to time after feeding (*p* < 0.01).

In relation to VFA concentration (Table 6), there was a tendency to increase total VFA (mM) and to reduce acetate molar proportion (% total VFA) (*p* < 0.10) with the increasing levels of CT. The molar proportion of propionate (%) was not affected, as well as the acetate:propionate ratio (*p* > 0.10). The molar proportion of butyrate (% of total) had a quadratic effect (*p* < 0.05) with CT dietary levels.

**Figure 2:**
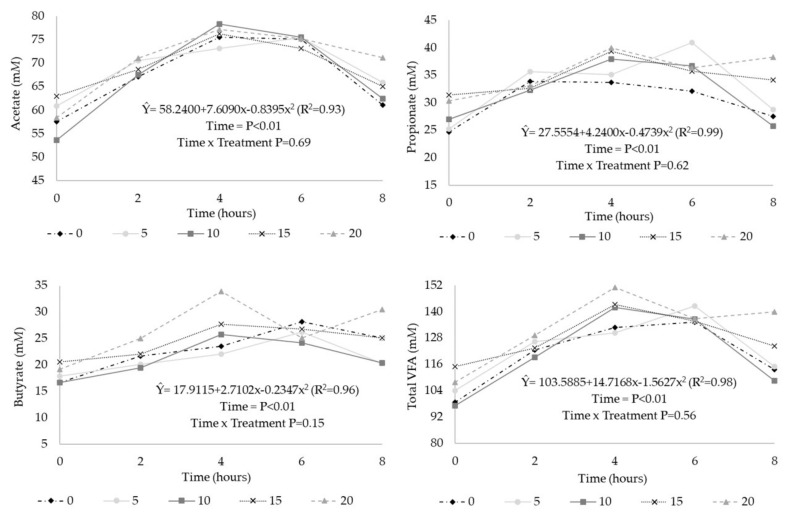
Concentration of ruminal volatile fatty acids in steers receiving diets with condensed tannins extract (CT) from *Acacia mearnsii*, as a function of time after feeding. 0 = no CT inclusion; 5 = 5 g of CT/kg of dry matter (DM); 10 = 10 g of CT/kg of DM; 15 = 15 g of CT/kg of DM; 20 = 20 g of CT/kg of DM.

In the evaluation of VFA as a function of time (Figure 2), the concentration of acetate, propionate, butyrate, and the total concentration of VFA (mM) had a quadratic effect (*p* < 0.01), with maximum points at 4.33, 4.28, 5.46, and 4.42 h after feeding, respectively. The acetate:propionate ratio was not affected by sampling time (*p* > 0.10). There was no interaction (*p* > 0.10) between time and treatment for these parameters.

## 4. Discussion

Condensed tannins are usually associated with a reduction in DM intake, depending on the amount ingested, the source used, structural chemistry, and molecular weight. This effect occurs due to the reduction in palatability and a reduction in nutrient digestibility [18,30,31]. However, in the present study, there was no reduction in the intakes of DM, OM, EE, CP, NDF, NFC, and TDN regardless of CT level used. Krueger et al. [32] evaluated CT of *A*. *mearnsii* to steers receiving 14.9 g/kg of DM in a finishing diet and did not observe effects on DMI. However, Grainger et al. [18] used CT from *A. mearnsii* in lactating cows’ diets with inclusion levels of 10.8 and 19.1 g/kg of diet DM and observed a reduction in DMI.

The absence of CT effects on the digestibility of DM and OM might be due to the levels used that might not have caused large changes in the ruminal microorganisms and thus did not reduce feed degradation; besides that, the DM intake was also not affected, so it was likely that levels up to 20 g/kg of dry matter did not affect palatability. Kozloski et al. [19] evaluated tannins of *A. mearnsii* at the levels of 20, 40, and 60 g/kg DM in sheep and observed a reduction in the digestibilities of DM, OM, NDF, and CP. Getachew et al. [33] performed an in vitro study with quebracho CT at 50, 100, and 150 g/kg of diet DM and observed lesser values for ruminal degradable protein in relation to the control treatment. Ahnert et al. [4] evaluated the infusion of CT from quebracho in cannulated steers at levels of 10, 20, 40, and 60 g/kg of DM and observed a reduction in the digestibilities of DM, OM, NDF, and ADF only at dosages greater than 40 g/kg, while the digestibility of CP was reduced even at the lowest dose. Another factor that may affect CT’s impact on digestibility may be related to ruminal volume and, therefore, CT concentration in the rumen; however, rumen volume is not commonly measured or reported.

The CP digestibility was reduced linearly with CT levels; this effect may be due to the binding effect of CT with protein [34]. In addition, it is possible that there was no dissociation of part of this complex (tannin-proteins) in the abomasum [30]; moreover, free tannins reaching the duodenum could inactivate intestinal enzymes or rebind to proteins [35], consequentially reducing protein digestibility. Similar results were observed by Aguerre et al. [9] with quebracho CT, with a linear reduction in the CP digestibility with levels of up to 18 g/kg of DM in diets of lactating cows; the authors also observed a reduction in the apparent digestibility of DM, OM, and NDF.

In relation to the ruminal protozoa population, the predominant genus was *Entodinium*, accounting for about 92% of total protozoa, corroborating with that observed in other studies [15,36]. However, there was no effect of CT on any of the evaluated genera; thus, the hypothesis of reducing the ruminal protozoa population with the use of CT has not been confirmed; this lack of effect could be influenced by the low dose of tannins used and by the adaptation of these microorganisms to the CT [15].

The results on CT and ruminal protozoa population are conflicting. Bhatta et al. [37] evaluated a mixture of hydrolyzable and condensed tannins and observed a reduction in the total protozoa population, while Benchaar et al. [38] evaluated the use of quebracho tannins at 6.40 g/kg DM and observed no effect on the number and generic distribution of ciliate protozoa. However, some studies with CT of *A. mearnsii* have observed a decrease in the Holotrich protozoa population [16,17].

The ruminal NH_3_-N contents were not influenced by the CT levels, demonstrating no effect of reducing ruminal protein degradation. These results corroborated with Gerlach et al. [39] who evaluated the effect of inclusion of CT of *A. mearnsii* with 9.20 and 29.6 g/kg of diet DM on dairy cows and also did not observe differences in ruminal ammonia concentration; however, Koenig et al. [40] evaluated CT extract from *A. mearnsii* (25 g/kg of DM) in steers on a high protein diet containing distillers grains and observed reduced ruminal NH_3_-N and ammonia emissions, indicating a reduction in protein degradation in the rumen. Similar results have also been obtained by other studies [9,41] evaluating CT of quebracho.

In the present study, the lack of effects on NH_3_-N and ruminal protozoa indicated that there might have been effects on protein metabolism after passage through the rumen since the apparent digestibility of the crude protein was reduced. Perna Junior et al. [17], using *A. mearnsii* CT at a dose of 6 g/kg in dry cows, also did not find differences in ruminal NH_3_-N. In the present study, NH_3_-N contents changed as a function of time, with maximum values approximately 2 h after feeding, corroborating with the results observed by Dickhoefer et al. [8], who evaluated quebracho tannins and observed the lowest NH_3_-N values from 4 to 8 h after feeding.

Regarding the ruminal pH, a linear reduction was observed in the time after feeding and with the inclusion levels of CT. The linear reduction of ruminal pH with CT inclusion levels might have occurred due to the low pH of the ingredient itself (4.9). However, even with this reduction, ruminal pH values, regardless of the CT level used, were in the range of 6 to 7, which is considered adequate to maintain the fibrolytic population and without ruminal acidosis, as indicated by Abdela [42]. Dickhoefer et al. [8] observed a reduction in ruminal pH with increasing doses of tannins, which related this effect to the lower pH values of the tannins and to the greater concentration of VFA with the highest doses of this compound.

The linear trend of increasing total VFA (mM) concentration with CT was contrary to the effect observed in some studies, in which the reduced rate of carbohydrate degradation was observed [4]. The similar results, as in the present study, were observed by Dickhoefer et al. [8]; the authors related the effect of increased VFA concentration to the reduction in water intake in the treatments with the inclusion of CT, rather than an effect on carbohydrate degradation.

The reduction of acetate concentration may be related to the effect of CT on ruminal fiber degradation. According to McSweeney et al. [43], cellulolytic bacteria are more sensitive to higher concentrations of CT compared to other microorganisms, and these compounds form complexes with carbohydrates of the plant cell wall [43] and can also cause a direct inhibition of CT in these microorganisms [44].

The increase in ruminal propionate concentration is desirable because the propionate formation pathway competes with CH_4_ synthesis [45] with possible improvements in animal performance and reductions in the environmental impact of animal production [16,37]. However, in the present study, there were no changes in the proportions of ruminal propionate. The quadratic effect of CT on butyrate proportion might have occurred due to changes in the microbial population and the reduction in acetate proportion since acetate can be converted to butyrate. Dickhoefer et al. [8] evaluated increased levels of CT from quebracho at levels up to 60 g/kg of diet DM and observed a linear increase in propionate and butyrate proportions in rumen fluid, while acetate proportions linearly decreased. The effects of supplementation with CT extracts on total VFA concentration and on the proportions of VFA have been inconsistent among studies, varying greatly, depending on the dose and source of CT [32,46].

## 5. Conclusions

The use of CT from black wattle (*A. mearnsii*) at levels up to 20 g/kg reduced the proportion of acetate, did not affect ruminal protozoa population, and reduced CP apparent digestibility. Based on these results, CT from black wattle is not recommended for improving nutrient utilization in steers at the tested levels. Further studies with different feeding strategies are required to further elucidate the potential roles of CT on ruminal fermentation and digestibility.

## Figures and Tables

**Table 1:** Chemical composition (g/kg dry matter) of ingredients used.

Item	Tifton 85 Hay	Ground Corn	Soybean Meal
Dry matter (g/kg of fresh matter)	850	907	889
Organic matter	920	985	925
Ether extract(EE)	16.7	20.0	12.0
Crude protein(CP)	113	82.7	479
Neutral detergent fiber	739	113	195
Non fiber carbohydrates (NFC) ^1^	110	782	302

^1^ Calculated according to the equation of Weiss et al. [20]: NFC = 100 − (CP + EE + ash) − NDFap.

**Table 2:** Ingredients and chemical composition of experimental diets in g/kg DM.

Ingredients	Levels of Condensed Tannins Inclusion (g/kg of DM)				
	0	5	10	15	20
Tifton 85 hay	600	600	600	600	600
Ground corn	337	328	322	314	307
Soybean meal	52.1	55.3	55.1	57.2	58.4
Mineral mix ^1^	10.0	10.0	10.0	10.0	10.0
Condensed tannin extract ^2^	-	6.20	12.4	18.7	24.9
Chemical Composition ^3^					
DM (g/kg of fresh matter)	868	866	868	868	873
OM	936	936	935	935	935
EE	19.3	17.9	19.9	20.9	20.4
CP	122	117	121	119	121
Estimated RDP ^4^	88.8	85.5	88.8	87.6	88.8
Estimated RUP ^4^	33.0	31.6	32.8	32.1	32.5
NDF	490	486	488	487	487
NDFap	447	444	444	443	444
NFC ^5^	348	358	349	351	350
Estimated TDN ^4^	648	642	637	631	626

DM: dry matter; OM: organic matter; EE: ether extract; CP: crude protein; RDP: rumen degradable protein; RUP: rumen undegradable protein; NDF: neutral detergent fiber; NDFap: corrected for ash and protein; NFC: non-fiber carbohydrates; TDN: total digestible nutrients; ^1^ Mineral mix: Ca: 130 g/kg; P: 65 g/kg; Mg: 5 g/kg; Co: 60 mg/kg; Mn: 1000 mg/kg; Zn: 3000 mg/kg; Se: 10 mg/kg; I: 65 mg/kg; S: 12 g/kg; Fe: 1200 mg/kg; Cu: 1000 mg/kg; Na: 120 g/kg. ^2^ Concentration of the commercial product: 805.2 g/kg of condensed tannins in the dry matter (Seta RC); ^3^ Analyzed chemical composition, unless otherwise specified; ^4^ Estimated with the NRC (2001) models; ^5^ Calculated according to the equations of Weiss et al. [18]: NFC = (100 − (CP + EE + MM + NDFap).

**Table 3:** Intake of dry matter and nutrients of steers receiving diets with increasing levels of condensed tannins extract from *Acacia mearnsii*.

Variables	Condensed Tannins Inclusion (g/kg of DM)					SEM	*p*-Value		
	0	5	10	15	20		CT	L	Q
DMI (kg/d)	8.90	8.25	9.00	8.42	8.75	0.324	0.17	0.87	0.47
DMI (g/kg BW)	11.8	10.9	12.0	11.0	11.5	0.447	0.13	0.67	0.59
OMI (kg/d)	8.34	7.73	8.42	7.88	8.19	0.301	0.17	0.83	0.46
CPI (kg/d)	1.07	1.01	1.10	1.04	1.07	0.040	0.25	0.81	0.76
EEI (kg/d)	0.17	0.15	0.18	0.18	0.18	0.011	0.10	0.08	0.70
NDFI (kg/d)	4.36	3.96	4.38	4.06	4.29	0.184	0.15	0.91	0.33
NDFI (g/kg BW)	5.79	5.25	5.86	5.34	5.65	0.249	0.11	0.75	0.40
NFCI (kg/d)	3.10	2.97	3.15	2.94	3.04	0.141	0.57	0.65	0.85
TDNI (kg/d)	6.92	6.42	6.93	6.59	6.86	0.293	0.33	0.95	0.39

DMI: dry matter intake; OMI: organic matter intake; CPI: crude protein intake; EEI: ether extract intake; NDFI: neutral detergent fiber intake; BW: body weight; NFCI: non-fiber carbohydrate intake; TDNI: total digestible nutrients intake; SEM: standard error of mean; CT: condensed tannins effect; L: linear; Q: quadratic.

**Table 4:** Apparent digestibility of dry matter and nutrients (%) in steers receiving diets with increasing levels of condensed tannins extract from *Acacia mearnsii*.

Variables	Condensed Tannins Inclusion (g/kg of DM)					SEM	*p*-Value		
	0	5	10	15	20		CT	L	Q
DMD	67.4	68.2	65.6	69.0	68.1	1.30	0.16	0.44	0.46
OMD	73.1	73.9	70.6	74.2	72.7	1.18	0.07	0.84	0.49
CPD ^1^	70.4	68.9	66.1	68.0	64.0	1.70	0.03	0.01	0.98
EED	69.5	67.3	72.3	76.0	72.9	3.00	0.11	0.04	0.71
NDFD	58.6	57.9	55.9	59.1	57.7	1.77	0.48	0.89	0.43
NFCD	85.8	86.1	84.2	85.5	87.0	0.964	0.14	0.40	0.05
TDN	79.4	78.3	78.0	80.0	79.3	0.979	0.35	0.30	0.49

DMD: dry matter digestibility; OMD: organic matter digestibility; CPD: crude protein digestibility; EED: ether extract digestibility; NDFD: neutral detergent fiber digestibility; NFCD: non-fiber carbohydrate digestibility; TDN: total digestible nutrients calculated according to the NRC [21]: TDN = dCP + dNFC + ((dEE − 1)×2.25) + dNDF − 7; SEM: standard error of mean; CT: condensed tannins effect; L: linear; Q: quadratic; ^1^ Ŷ = 69.9086 − 2.7284x (R^2^ = 0.75).

**Table 5:** Protozoa population in ruminal content (*n* × 10^6^/mL of ruminal content) in steers receiving diets with increasing levels of condensed tannins extract from *Acacia mearnsii*.

Item	Condensed Tannins Inclusion (g/kg of DM)					SEM	*p*-Value		
	0	5	10	15	20		CT	L	Q
*Entodinium*	1.21	1.03	1.10	1.08	0.86	0.120	0.13	0.03	0.51
*Dasytricha*	0.04	0.02	0.05	0.04	0.02	0.012	0.15	0.68	0.34
*Isotricha*	0.03	0.02	0.02	0.02	0.02	0.006	0.10	0.05	0.25
*Charonina*	0.01	0.02	0.02	0.01	0.01	0.007	0.43	0.19	0.45
*Eremoplast*	0.01	0.01	0.01	0.01	0.01	0.005	0.78	0.30	0.59
*Metadinium*	0.01	0.01	0.01	0.00	0.01	0.004	0.84	0.81	0.95
Total	1.31	1.11	1.20	1.18	0.93	0.005	0.12	0.03	0.45

SEM: standard error of mean; CT: condensed tannins effect; L: linear; Q: quadratic.

**Table 6:** Ruminal pH, ammonia nitrogen (N-NH_3_), and volatile fatty acids in steers receiving diets with increasing levels of condensed tannins extract from *Acacia mearnsii*.

Variables	Condensed Tannins Inclusion (g/kg DM)					SEM	*p*-Value		
	0	5	10	15	20		CT	L	Q
pH ^1^	6.75	6.73	6.67	6.59	6.57	0.040	<0.01	<0.01	0.87
N-NH_3_ (mg/dL)	11.0	12.1	11.1	11.3	11.3	1.02	0.86	0.90	0.78
Total VFA (m*M*) ^2^	121	124	122	128	133	5.11	0.09	0.01	0.36
Acetate (%) ^3^	56.0	56.3	56.3	54.0	53.5	1.08	0.02	<0.01	0.22
Propionate (%)	24.9	26.5	26.1	26.8	26.7	0.91	0.17	0.23	0.61
Butyrate (%) ^4^	19.0	17.1	17.4	19.1	19.6	1.28	0.01	0.07	<0.01
Acetate:propionate	2.31	2.17	2.13	2.04	2.06	0.095	0.17	0.02	0.41

Total VFA (mM): sum of acetic, propionic, and butyric acids; SEM: standard error of mean; CT: condensed tannins effect; L: linear; Q: quadratic; ^1^ Ŷ = 6.76264 − 0.01011x (R^2^ 0.89); ^2^ Ŷ = 120.5416 + 0.5549x (R^2^ = 0.83); ^3^ Ŷ = 56.6789 − 0.1463x (R^2^ = 0.68); ^4^ Ŷ = 18.7080− 0.3164x + 0.0192x^2^ (R^2^ = 0.95).
